# Hemi-Ultrathin Descemet Stripping Automated Endothelial Keratoplasty (Hemi-UT-DSAEK) Using Pediatric Donor Corneas: A Case Series

**DOI:** 10.3390/jcm12175442

**Published:** 2023-08-22

**Authors:** Pia Leon, Lorena Francescutti, Pietro Gentile, Federica Birattari, Diego Ponzin, Davide Camposampiero, Antonella Franch, Mohit Parekh

**Affiliations:** 1 Department of Ophthalmology, SS Giovanni e Paolo Hospital, 30122 Venice, Italy; 2 International Center for Ocular Physiopathology, Fondazione Banca degli Occhi del Veneto Onlus, Zelarino, 30174 Venice, Italydiego.ponzin@fbov.it (D.P.);; 3 Schepens Eye Research Institute, Mass Eye and Ear, Boston, MA 02114, USA; 4 Department of Ophthalmology, Harvard Medical School, Boston, MA 02115, USA

**Keywords:** UT-DSAEK, eye bank, cornea, endothelium, FECD, hemi-UT-DSAEK, pediatric donors

## Abstract

Objective: We sought to evaluate the clinical outcomes of hemi-UT-DSAEK grafts from the pediatric donor corneas of patients affected by Fuchs Endothelial Corneal Dystrophy (FECD). Methods: A prospective, interventional case series was conducted at the Ophthalmology Department of Venice Civil Hospital and the Veneto Eye Bank Foundation (Venice, Italy). Six eyes of six patients affected by FECD received large-diameter, semicircular hemi-UT-DSAEK grafts obtained from three pediatric donor corneas using the standard pull-through method. Endothelial cell density (ECD), central corneal thickness (CCT), best-corrected visual acuity (BCVA) and intraoperative and postoperative complications were recorded at different time intervals up to 12 months. Results: The average donor age was 64.6 ± 8.6 years, and the pre-operative ECD was 3266 ± 225 cells/mm^2^. At 12 months postoperatively, the average ECD was 1376 ± 509 cells/mm^2^ with a mean decrease of 56.8 ± 19.1% from the preoperative donor count. At 12 months, four out of six eyes had significantly improved and reached a BCVA of ≥20/25 (Snellen equivalent). The mean CCT significantly decreased from 788 ± 138 μm before surgery to 576 ± 30 μm at 12 months postoperatively (*p* < 0.01). Conclusions: Hemi-UT-DSAEK grafts using pediatric donor corneas are surgically feasible and can provide similar clinical outcomes compared to conventional UT-DSAEK. Transplanting pediatric donor tissues with high ECD into two patients could potentially increase the donor tissue pool to treat endothelial disease.

## 1. Introduction

Descemet Stripping Automated Endothelial Keratoplasty (DSAEK) is the most frequently performed subtype of endothelial keratoplasty for the treatment of Fuchs Endothelial Corneal Dystrophy (FECD) [1]. In recent years, innovative techniques such as ultrathin DSAEK (UT-DSAEK) and Descemet Membrane Endothelial Keratoplasty (DMEK) have been developed, providing superior visual outcomes with thinner corneal grafts [2,3]. The major limitations of these procedures include graft dislocation, which can be reduced with the advancement of surgical techniques and endothelial cell loss (ECL) over time, which is still inevitable [3,4]. Therefore, the availability of donor tissues with a high endothelial cell density (ECD) is crucial for endothelial keratoplasty [5,6,7,8]. Previous studies reported a significant difference in the relationship between age and ECD [9,10]; the younger the donor, the higher the endothelial cell count in the graft [5,6,7,8]. However, the age of the donor may influence the surgical outcome, since tissues from younger donors are more elastic in nature, have a steeper curvature and can thus be relatively difficult to handle [5,6,7]. Currently, only the circular central portion (full-moon-shaped graft) of the donor tissue is harvested and transplanted via EK procedures. However, there is growing evidence that in eyes affected by FECD, different graft shapes can be used to obtain corneal clearance [11]. Lam et al., in 2014, presented a prospective, interventional case series of three eyes affected by FECD that received semicircular, large-diameter hemi-DMEK grafts [12], while Birbal et al., in 2018, showed the extended clinical results of 10 eyes that underwent hemi-DMEK, with up to 4 years of follow-up [11]. Most recently, Moloney et al. presented the safety and efficacy of descemetorhexis without endothelial keratoplasty (DWEK) to obtain clear corneas in central endothelial pathologies, where the presence of healthy peripheral endothelial cells may allow successful corneal repopulation and recompensation, without the need for donor tissues [13]. As the ECD in pediatric corneas is extremely high compared to adult corneas, therefore, its usage must be optimized [14]. The purpose of the present study is thus to evaluate the clinical outcomes of large-diameter, semicircular (half-moon-shaped), UT-DSAEK grafts from pediatric donor corneas of patients affected by FECD. The advantage of hemi-UT-DSAEK over circular UT-DSAEK is that two grafts can be obtained from one donor cornea with high ECD and transplanted into two different recipients. This technique could potentially double pediatric donor tissue usage and shorten the waiting list for DSAEK.

## 2. Patients and Methods

Six eyes of six patients affected by stage II FECD were involved in this prospective, interventional case series. The present study was conducted according to the Declaration of Helsinki and was approved by the local ethics committee. All patients provided written informed consent before surgery.

One cornea from a 3-year-old donor and two corneas from a 10-year-old donor were used. The donor consent was obtained from the parents. Graft tissues were prepared at the Veneto Eye Bank Foundation (Venice, Italy) using the microkeratome-assisted double-pass technique, as previously described by Busin et al. [2]. Briefly, the donor cornea was mounted on an artificial anterior chamber, and the central corneal thickness (CCT) was measured using an anterior segment optical coherence tomography machine (AS-OCT; SS-1000 Casia; Tomey Corporation, Nagoya, Japan). An initial cut was performed using a moria microkeratome with a 300 µ head. Based on the thickness obtained from the first cut, a second cut was performed after turning the AAC by 180°, starting from the opposite end of the first cut. The pressure was standardized by raising and clamping the infusion bottle 120 cm above the AAC [2].

All surgical procedures were performed by a single expert surgeon (P.L.) at the Ophthalmology Department of Venice Civil Hospital (Venice, Italy) between April 2021 and July 2021. The peripheral cornea was clear in all cases, and the central area affected by guttae was greater than 5 mm in diameter; hence, the patients were not suitable for descemetorhexis without endothelial keratoplasty (DWEK) procedure. Two eyes were pseudophakic, and out of the remaining four phakic eyes, three underwent combined phacoemulsification, intraocular lens implantation and endothelial keratoplasty. A complete descemetorhexis was performed in all the eyes.

In the operating room, the pre-cut corneoscleral buttons were divided into two equal halves with a surgical diamond knife and implanted, following a standard pull-through technique, with a modified Busin glide. While the anterior chamber was maintained with fluid, each graft was carefully oriented with the widest diameter along the horizontal meridian. An air bubble was then injected into the anterior chamber underneath the graft to keep it pressed against the recipient stroma. The anterior chamber was completely filled with air for up to 120 min, and the patients were instructed to lie in the supine position. All the patients were examined two hours after surgery using the slit-lamp examination, and some air was removed, leaving the eye at 50% minimal residual air. Postoperative management included antibiotic (netilmicin 0.3%) and steroid (dexamethasone 0.1%) eye drops six times a day for three months with a slow tapering off over a period of six months, and the treatment was stopped after six months. Patients underwent a follow-up ophthalmic examination at 1 day, 1 week and 1, 3, 6, 9 and 12 months postoperatively. Each follow-up included a slit-lamp examination, best-corrected visual acuity assessment (BCVA), endothelial biomicroscopy (Specular Microscope CEM-530, Nidek, San Jose, CA, USA), anterior-segment optical coherence tomography (AS-OCT) and pachymetry (AS-OCT MS-39, CSO, Florence, Italy). The postoperative ECD was calculated centrally in the recipient patient’s cornea from the equivalent paracentral area of the hemi-UT-DSAEK graft. The later time points were compared with the pre-surgical/donor values using Friedman’s repeated-measure non-parametric test with Dunn’s post hoc test (significance level alpha = 0.05; 95% CI) to obtain statistical significance.

## 3. Results

The donor graft thickness, after preparing the UT-DSAEK graft, was found to be in the range of 87–130 μm with a diameter of 8.75 mm (*n* = 1) and 9.00 mm (*n* = 2). The peripheral cornea was clear in all the cases, as we included only grade II FECD patients. The preoperative mean ECD of the peripheral cornea was 1853 ± 452 cells/mm^2^. The postoperative ECD was calculated centrally in the recipient patient’s cornea from the equivalent paracentral area of the hemi-UT-DSAEK graft.

**Figure 1:**
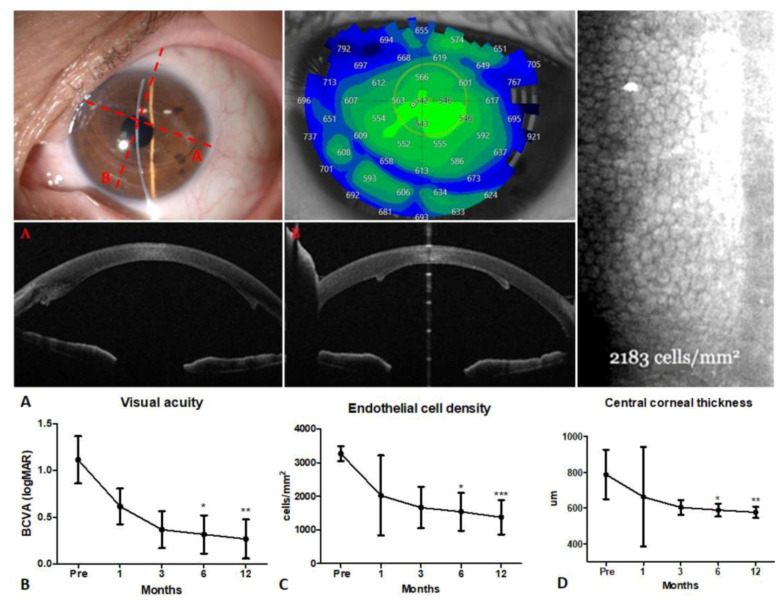
Clinical outcomes of hemi-UT-DSAEK using pediatric tissue transplanted into the left eye of patient 2. (**A**) Slit-lamp, pachymetry, specular microscopy and AS-OCT at the 12-month follow-up visit. (**B**) Best-corrected visual acuity (logMAR), (**C**) endothelial cell density (cells/mm^2^) and (**D**) central corneal thickness (µm) of patients at baseline, (pre) and at 1, 3, 6 and 12 months. Letters (A and B) marked in red indicate the orientation of the graft. * *p* < 0.05; ** *p* < 0.01; *** *p* < 0.001.

At the time of surgery, the mean ± standard deviation patient age was 64.6 ± 8.6 years (range 51–78 years). After surgery, all the grafts were well-centered and completely attached (Figure 1A). One eye required a rebubbling procedure for graft detachment one week after the surgery, and one eye presented ocular hypertension one week after surgery, which was successfully treated with topical hypotensive therapy eye drops (timolol 0.5%) twice a day. The postoperative course was uneventful in all other cases. All the eyes showed improved corneal transparency at 1 month postoperatively, which appeared to be stable at 12 months.

BCVA improved in all cases at 12 months postoperatively, and four out of six eyes reached a BCVA of ≥20/25 (Snellen equivalent). One of the two remaining eyes underwent a rebubbling procedure for graft detachment and experienced an improvement in BCVA from 20/200 to 20/32 (Snellen equivalent), while the other eye remained stable (20/400, Snellen equivalent) due to pre-existing glaucoma. Overall, BCVA significantly improved from 1.1 ± 0.6 logMAR (presurgical) to 0.32 ± 0.5 logMAR at 6 months (<0.05) and 0.27 ± 0.5 logMAR at 12 months (*p* < 0.01) (Figure 1B). The donor ECD significantly decreased from 3266 ± 225 cells/mm^2^ (range 3000–3500 cells/mm^2^) preoperatively to 1538 ± 565 cells/mm^2^ at 6 months (*p* < 0.05) and 1376 ± 510 cells/mm^2^ at 12 months (*p* < 0.001) after surgery (Figure 1C). The mean endothelial cell loss (ECL) from the preoperative donor count was 35.3 ± 27.8%, 51.8 ± 21.0% and 56.8 ± 19.1% at 1, 6 and 12 months postoperatively, respectively. The central corneal thickness (CCT) decreased from 789 ± 139 μm before surgery to 663 ± 71 μm at 1 month and significantly to 589 ± 36 μm at 6 months (*p* < 0.05) and 577 ± 31 μm at 12 months postoperatively (*p* < 0.01) (Figure 1D) (Table 1) (Supplementary Figures S1 and S2).

## 4. Discussion

The concept of hemi-DMEK was introduced in 2014 by Lam et al. [12], when the authors suggested using a centralized half-moon-shaped graft, as there is no optical or technical reason to use the entire central portion alone. In fact, hemi-DMEK could be produced through slight modifications without any tissue loss. However, it is relatively difficult to obtain a DMEK graft consistently from young donors with a high endothelial count; hence, UT-DSAEK has become a favored choice in such cases. In 2011, Neff et al. [15] reported that DSAEK grafts thinner than 131 µm show better results than DMEK grafts. Confounding reports have been observed, indicating suboptimal final visual acuity after DSAEK due to the presence of stromal interface [16,17,18]. DMEK, on the other hand, has shown consistent improvement in terms of visual rehabilitation and outcomes, with patients achieving 20/20 vision and a reduced rate of immunologic rejection [19,20,21]. However, although attempts have been made to standardize DMEK graft preparation [22], several challenges limit the use of DMEK, including manipulation, implantation, the rebubbling rate and the feasibility of using DMEK in complicated eyes. In this study, the graft was positioned along the longest horizontal meridian, which was followed in our study to ensure that the unfolded graft, if used, would be centered precisely. Pediatric donor tissues must be used optimally, as they have a high endothelial cell count but can be difficult to handle if pursued for DMEK due to its elasticity. Hence, UT-DSAEK could be advantageous in such cases. Hemi-DMEK and UT-DSAEK grafts have shown favorable visual outcomes; hence, we combined both techniques to investigate whether the pediatric donors could be used to optimize the use of these precious tissues.

Following hemi-DMEK, Birbal et al. reported the restoration of the host cornea to its physiological thickness as early as 1 month. Corneal thickness reduced from 745 um (pre) to 533 um at 1 year postoperatively [11], whereas Lam et al. reported a CCT of 527 um at 6 months. The average CCT in our study decreased from 789 before surgery to 663 μm at 1 month, 589 μm at 6 months (*p* < 0.01) and 577 μm at 12 months postoperatively (*p* < 0.001), showing similar results to hemi-DMEK. The results following DMEK are expected to be homogeneous due to the uniform thickness of the graft; however, we did not find any difference in the outcome measures following the UT-DSAEK transplants even when the graft thickness ranged from 87 to 138 µm (Table 1). The denuded stromal region adjacent to the hemi-UT-DSAEK graft showed clearance between 9 and 12 months (Supplementary Figures S1 and S2).

Although corneal grafts from younger donors are more difficult to handle, hemi-UT-DSAEK with grafts obtained from pediatric donors are technically feasible and provide higher ECD than grafts from adult corneas. However, it is recommended to maintain consistent pressure in the artificial anterior chamber during UT-DSAEK preparation to avoid any perforations. In the present study, the preoperative mean graft ECD was 3266 ± 225 cells/mm^2^ (range 3000–3500 cells/mm^2^), whereas the general healthy adult ECD ranges are between 2000 and 3000 cells/mm^2^ [23]. The grafts were prepared at the eye bank using standardized procedures and properly positioned using the classic UT-DSAEK technique during surgery. The goal of hemi-UT-DSAEK flap placement is to perfectly cover the optic zone. The horizontal or oblique implant position is equivalent for the purpose of endothelial tissue replacement in patients affected by FECD. Therefore, the graft was oriented with the widest diameter along the corneal horizontal meridian, as previously described by Birbal et al. for hemi-DMEK [11].

The use of hemi-DMEK grafts with larger diameters has been reported previously [12]. Romano et al. reported that larger DMEK grafts (9.5 mm), similar to larger UT-DSAEK grafts, were easier to handle and did not incur any significant ECL, which can be useful for long-term graft survival [24]. Although a higher rebubbling rate has been reported following a large-diameter DMEK graft transplant, the long-term survival has been shown to be around 93%. However, in our study, we did not observe any graft detachment. Hence, a larger-diameter graft with a high ECD could be used for hemi-UT-DSAEK.

A 12 postoperative months, the ECD showed a mean decrease of 56.8% from the preoperative donor count, most of which (35.3%) occurred in the first month. Lam et al. showed a similar decrease after hemi-DMEK (31–49%) in the initial months [12]. The mean ECL after a conventional DMEK is approximately 30–35% at 6 months [19,25], where again, a steep decrease in the ECD is found during the first month. The mean ECD decrease after standard UT-DSAEK is reported to be approximately 29%, 33% and 35% at 3 months, 6 months and 12 months postoperatively, respectively [2]. However, ECLs of approximately 66% at 6 months [26] and 69% at 12 months [11], after transplanting hemi-DMEK grafts from adult donors (average age, 70 years) with a lower ECD (with average of 2730 cells/mm^2^) than pediatric corneas, have been reported previously. In our case series, the initial decrease in ECD within the first month was sharper than that of standard UT-DSAEK, but it may be compensated by the higher ECD of younger donor corneas [27]. Lam et al. also suggested that there are variations in cell migration between hemi- and conventional DMEK. A different migratory pattern of the cells can be expected due to the semicircular shape of the graft with a larger denuded area of the stromal bed and the involvement of stromal interface. This leaves a larger gap, and the endothelial cells may take a two-way approach (centripetal from the host and centrifugal from the donor) to repopulate in the donor graft [12]. However, this hypothesis needs to be investigated further.

In terms of visual outcomes, Birbal et al. described a BCVA of ≥20/40 in 86% of patients following hemi-DMEK, which remained stable for 2 years and improved thereafter [11]. Lam et al. [12] reported a BCVA of 20/22, 20/40 and 20/17 in their three cases in the 12-month postoperative period, following hemi-DMEK [28]. A larger sample study of 56 patients by Romano et al. reported a significant improvement in BCVA after transplanting preloaded DMEK compared with preloaded UT-DSAEK at the end of 1 year. However, the rate of rebubbling with preloaded DMEK was 44% compared to that for preloaded UT-DSAEK, which was found to be 12.9% [29]. UT-DSAEK has shown BSCVA of 20/20 in approximately 12% of cases and at least 20/40 in 64% of cases with continuous improvements over time, reaching 20/20 vision in approximately 49% of cases at 2 years. Approximately 77% of cases (phakic) were shown to reach a BSCVA of at least 20/20 as early as 6 months after UT-DSAEK [30]. UT-DSAEK and DMEK have shown similar logMAR BSCVA curves throughout the entire follow-up period compared to conventional DSAEK [21,31]. Although visual rehabilitation after UT-DSAEK is slower compared to DMEK, the percentage of eyes with BSCVA of 20/20 or better is roughly identical as early as 1 year postoperatively [30].

The visual outcome after UT-DSAEK indicates that the use of thin grafts allows a higher number of eyes to achieve early visual rehabilitation. However, the percentage of eyes recovering 20/20 vision after UT-DSAEK is lower [19,21,32,33], indicating that other factors, in addition to a stromal interface, may be responsible for the final visual outcome. Patel et al. showed a small peak in corneal backscatter initiating from the posterior stroma of the recipient cornea, speculated to be correlated with long-standing edema, as well as anatomical and functional changes [34].

Graft survival probability following UT-DSAEK grafts has been shown to be roughly 98% and 96% at 12 and 24 months [31]. Price et al. reported a 93% survival rate after DSAEK at 5 years [35], with a significantly higher value of survival for Fuchs patients (95%). One-year graft survival for DSAEK, excluding the initial learning curve of surgeons, has been reported to vary between 94 and 100% [30]. Our study was limited to 12 months, but long-term outcomes and graft survival analysis would be crucial for hemi-UT-DSAEK grafts from pediatric donors.

Following hemi-DMEK, Birbal et al. reported a rebubbling rate of 40% with complications such as persistent graft detachment (*n* = 1), secondary graft failure (*n* = 1) at 2.5 years and suspected allograft reaction after 1.5 years (*n* = 1) postoperatively [11], whereas, Lam et al. reported no immediate postoperative complications following hemi-DMEK [28]. Although we did not find any intraoperative complications in our study, the postoperative course was uneventful in all but two cases, which showed rebubbling (*n* = 1) and ocular hypertension (*n* = 1) one week after surgery, which were successfully treated.

Despite the limited sample size of our case series, the clinical outcomes of hemi-UT-DSAEK using pediatric donor corneas are encouraging, especially as they are comparable with standard UT-DSAEK. Transplanting a hemi-UT-DSAEK graft is surgically feasible and could potentially increase the availability of donor tissues for endothelial keratoplasty. Experienced surgeons may accept grafts from younger donors with confidence and consider performing hemi-UT-DSAEK. In fact, these grafts can also be prepared by eye banks and shipped as preloaded tissues to further enhance their quality and validation [36], in addition to reducing tissue wastage and saving time in surgery. Optimizing young donors by utilizing one graft for multiple recipients with a high ECD would be advisable [37]. This study could also help to counter the common stigma that younger donors cannot be used for EK. While age may make a difference for DMEK, it does not have a significant impact on DSAEK or UT-DSAEK.

## Figures and Tables

**Table 1:** Overview of the donor data and demographic, baseline and follow-up period characteristics of the patients.

Patient Data										Donor Data								
Patient No.	Sex		Age at Surgery (y)	Eye	Lens Status	Concomitant Eye Disease			Combined Phaco + IOL	Age at Death (y)		Cause of Death	Death- Harvesting Time Interval (min)	ECD (Cells/mm^2^)			CCT (μm) (UT-DSAEK Graft)	
1	M		51	R	P	none			no	3		Neoplasia	216	3000			138	
2	F		65	L	P	none			yes									
3	M		69	R	P	DME			yes	10		Trauma	168	3300			90	
4	F		63	L	P	none			yes									
5	F		62	R	PP	none			no	10		Trauma	168	3500			87	
6	M		78	R	PP	Glaucoma			no									
Mean ± SD			64.7 ± 8.9											3266.7 ± 225.1			105 ± 25.6	
	**Preoperative**			**Intraoperative**	**BCVA (Snellen)**					**ECD (Cells/mm^2^) [ECL (%)]**				**CCT (μm)**				
Patient No.	BCVA (Snellen)	CCT (μm)		Graft position	1M	3M	6M	12M		1M	3M	6M	12M	1M	3M	6M		12M
1	20/25	650		H	20/25	20/25	20/20	20/20		2360 [21.3]	2060 [31.3]	1869 [37.7]	1840 [38.7]	614	613	605		580
2	20/200	630		O	20/100	20/25	20/20	20/20		2900 [3.3]	2750 [8.3]	2539 [15.4]	2183 [27.2]	765	553	531		542
3	20/200	738		O	20/200	20/63	20/50	20/32		NE	1350 [59.1]	1203 [63.5]	1110 [66.4]	NE	634	614		600
4	20/400	867		O	20/32	20/25	20/25	20/20		1532 [53.6]	1290 [60.9]	1210 [63.3]	1173 [64.5]	570	564	557		550
5	20/400	866		O	20/50	20/25	20/25	20/25		1300 [62.9]	1150 [67.1]	1062 [69.7]	950 [72.9]	692	633	605		590
6	20/2000	982		H	20/400	20/400	20/400	20/400		NE	1380 [60.1]	1342 [61.7]	1002 [71.4]	750	640	622		610
Mean ± SD		788.8 ± 138.9								2023 ± 740.8 [35.3 ± 27.8]	1663.3 ± 619.6 [47.9 ± 23.1]	1537.5 ± 565 [51.8 ± 21.0]	1376.3 ± 509.9 [56.8 ± 19.1]	678.2 ± 84.7	606.2 ± 38.2	589 ± 36.4		578.7 ± 27.3

Graft position: H, long graft margin oriented horizontally; O, long graft margin oriented obliquely; M, male; F, female; y, years; R, right; L, left; P, phakic; PP, pseudophakic; DME, diabetic macular edema; ECD, endothelial cell density; ECL, endothelial cell loss; BCVA, best-corrected visual acuity; CCT, central corneal thickness; 1M, 1-month follow-up visit; 3M, 3-month follow-up visit; 6M, 6-month follow-up visit; 12M, 12-month follow-up visit; NE, not evaluable. Patient 3 experienced a graft detachment and underwent a rebubbling procedure after two weeks following primary surgery. Patient 5 experienced postoperative ocular hypertension.

## Data Availability

The table includes all the raw data available in this study.

## Supplementary Materials

The following supporting information can be downloaded at: https://www.mdpi.com/article/10.3390/jcm12175442/s1, Figure S1: Slit lamp images of the left eye of patient 2 before and after hemi-UT-DSAEK tissue transplant using pediatric tissues at their respective follow-up time points; Figure S2. Corneal thickness measurements of the left eye of patient 2 at different time points after hemi-UT-DSAEK tissue transplant using pediatric tissue.
