# Supplementary material for: Hemi-Ultrathin Descemet Stripping Automated Endothelial Keratoplasty (Hemi-UT-DSAEK) Using Pediatric Donor Corneas: A Case Series
Source: J Clin Med. 2023 Aug 22;12(17):5442. doi: 10.3390/jcm12175442 (PMC10487646; doi:10.3390/jcm12175442)
Supplement: Supplementary file 1 [file jcm-12-05442-s001.zip › jcm-2479414-supplementary.pdf]

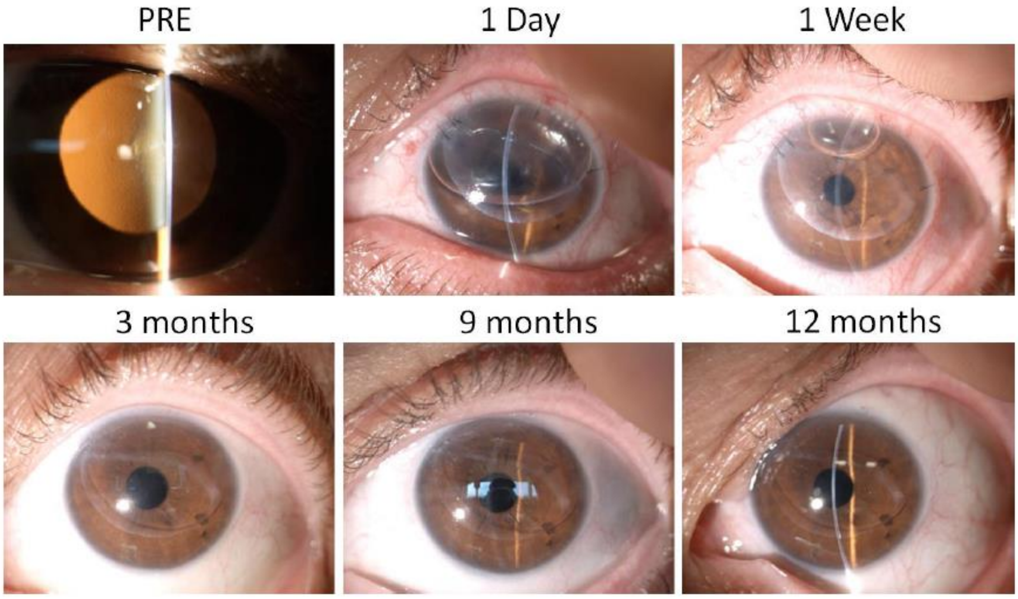

Figure S1. Slit lamp images of the left eye of patient 2 before and after hemi-UT-DSAEK tissue transplant using pediatric tissues at their respective follow-up time points.

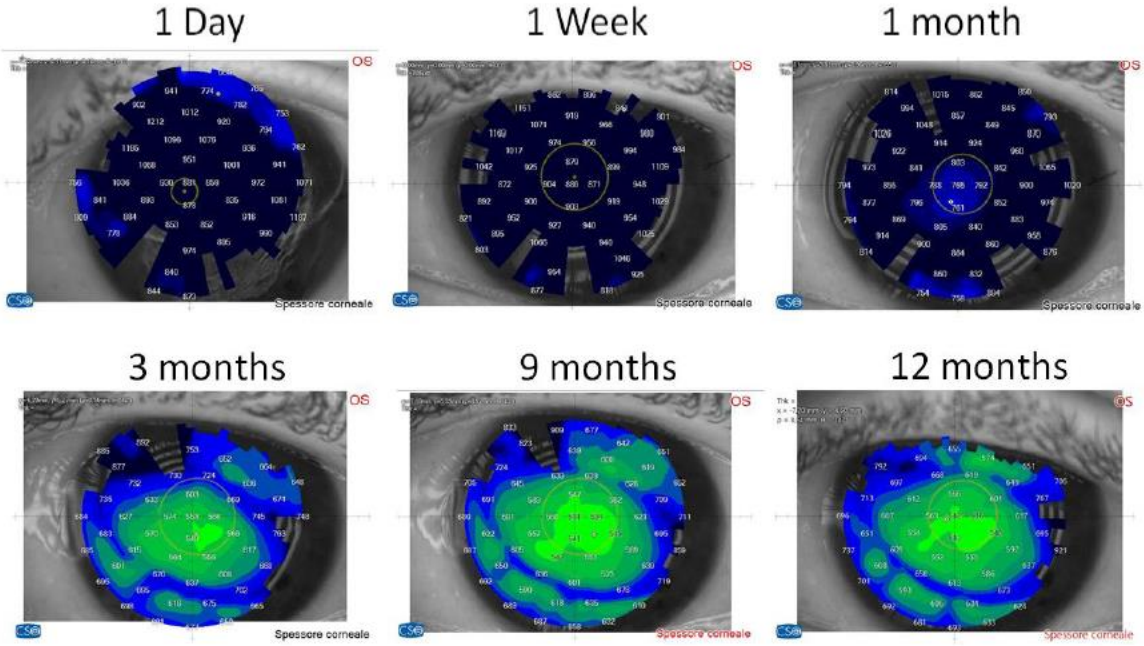

Figure S2. Corneal thickness measurements of the left eye of patient 2 at different time points after hemi-UT-DSAEK tissue transplant using pediatric tissue.
